# Genomic Selection for Milk Yield and Milk Composition Traits in Dairy Goats Using Machine Learning and Prior-Information Models

**DOI:** 10.3390/ani16152426

**Published:** 2026-08-05

**Authors:** Jianqing Zhao, Wei Wang, Jiayidaer Kamalibieke, Yuanpan Mu, Jun Luo

**Affiliations:** 1 College of Animal Science, Xinjiang Agricultural University, Urumqi 830052, China; zhao2021@nwafu.edu.cn; 2 Shaanxi Key Laboratory of Molecular Biology for Agriculture, College of Animal Science and Technology, Northwest A&F University, Xianyang 712100, China

**Keywords:** dairy goat, genomic selection, machine learning, low-coverage whole-genome sequencing, prior information

## Abstract

Genomic selection (GS) provides an effective approach to accelerating genetic gain in dairy goats, but the prediction performance is strongly influenced by the statistical model, marker density, phenotype adjustment strategy, and biological architecture of the target trait. In this study, dairy goat populations comprising Xinong Saanen and Saanen dairy goats from major production regions in China were used to evaluate genomic prediction for milk yield (MY), milk fat percentage (MFP), and milk protein percentage (MPP). Genotypes from 1034 dairy goats were generated using low-coverage whole-genome sequencing (lcWGS), imputed to improve genotype completeness and accuracy; a high-quality chip-based dataset was also constructed from previously developed 25K single-nucleotide polymorphism (SNP) chip loci. Conventional genomic best linear unbiased prediction (GBLUP) models, Bayesian regression models, and machine learning algorithms were compared using 10-fold cross-validation. Bayesian models showed clear trait-specific advantages, with BayesB improving MFP prediction by approximately 12.9% relative to GBLUP under the 25K chip-based strategy. Among machine learning methods, gradient boosting models performed strongly; extreme gradient boosting (XGBoost) improved the prediction accuracy for MY, MFP, and MPP by 14.3%, 17.9%, and 18.5%, respectively, relative to GBLUP under the chip-based strategy. Incorporating genome-wide association study (GWAS)-derived prior information and selection signature priors further improved the prediction accuracy, particularly for milk composition traits. Overall, the results indicate that genomic prediction in dairy goats can be optimized by matching models, genotyping platforms, and prior biological information to the genetic characteristics of the target trait.

## Simple Summary

Improving milk yields and milk composition is a central objective of dairy goat breeding. This study compared conventional genomic prediction models, Bayesian regression models, and machine learning algorithms for three economically important traits in dairy goats: milk yield, milk fat percentage, and milk protein percentage. The results showed that the prediction accuracy depended jointly on the trait, genotyping strategy, statistical model, and type of biological prior information. Bayesian and gradient boosting methods were particularly useful for milk composition traits, whereas chip-based genotyping remained cost-effective for milk yield. These findings provide a practical framework for integrating machine learning, low-coverage whole-genome sequencing, SNP chip data, GWAS signals, and selection signature information into genomic selection programs for dairy goats.

## 1. Introduction

The dairy goat industry is expanding rapidly, accompanied by a sustained increase in market demand. Improving key economic traits, including milk yield, milk quality, and reproductive performance, is therefore essential for promoting efficient and sustainable development of the industry [1,2]. Conventional selection mainly relies on phenotypes and pedigree records. However, genetic evaluation based on these data can be affected by environmental variation, limiting the rate of genetic gain [3]. In French dairy goats, conventional BLUP-based prediction has even shown lower accuracy than parent-average prediction in some cases [4]. Genomic selection, which integrates genome-wide marker information, offers a powerful tool for more precise breeding [5].

The core of genomic selection is the construction of efficient statistical prediction models. Early studies often applied single-step genomic best linear unbiased prediction (ssGBLUP), which combines pedigree, phenotypic, and genotypic data to improve the prediction accuracy [6]. Nevertheless, conventional models usually assume linear and independent marker effects, which may be insufficient in characterizing the genetic architectures of complex traits. In French dairy goats, protein content is partly regulated by the major αs1-casein locus; conventional ssGBLUP increased the prediction accuracy by only 5–7% for this type of trait, whereas weighted ssGBLUP achieved an additional improvement of approximately 6% by optimizing marker weights [7]. Similarly, although single-breed reference panels can achieve genotype imputation accuracy above 80%, their performance is reduced in across-breed applications [8]. These limitations highlight the need for model optimization.

In recent years, machine learning methods have provided new opportunities for genomic selection. Kernel ridge regression (KRR) and AdaBoost.RT have been reported to improve the prediction accuracy for udder traits in dairy goats by 11–20% compared with GBLUP, while also enhancing model stability [9]. Deep learning approaches can capture latent non-linear patterns in genomic data through multilayer networks and are particularly suitable for modeling non-additive genetic effects. Moreover, multi-omics integration, including the combined use of GWAS, transcriptomics, and metabolomics, can systematically characterize the regulatory networks underlying milk composition traits [1]. Functionally prioritized variant screening strategies can further improve the targeting efficiency and accuracy of genomic prediction [10].

Applying machine learning to genomic selection for economically important dairy goat traits may overcome the limitations of conventional statistical models and enable more accurate evaluations of genetic potential. By integrating prior biological information and using advanced predictive algorithms, it is possible to characterize the genetic mechanisms of dairy goat production traits more comprehensively and to support practical breeding decisions. The objective of this study was to compare the performance of multiple statistical and machine learning models for genomic selection in dairy goats and to evaluate the effects of GWAS and selection signature prior information on prediction accuracy.

## 2. Materials and Methods

### 2.1. Population, Phenotypes, and Genotypes

The study populations comprised 1034 dairy goats, including 753 Saanen dairy goats and 281 Xinong dairy goats, sampled from major production areas, including Shaanxi, Zhejiang, and Inner Mongolia, China. The final genomic prediction dataset included all 1034 dairy goats after sample-level quality control. Phenotypic records were systematically collected across growth and production stages according to the technical specifications of the Third National Survey of Livestock and Poultry Genetic Resources in China. Based on industry demand, three core economic traits were selected for genomic selection analysis: milk yield (MY), milk fat percentage (MFP), and milk protein percentage (MPP). Breed, region, herd, parity, age, season or year of recording, and lactation-stage/test-day information were considered as fixed effects when available. To avoid confounding by major environmental effects, the phenotypes used for genomic prediction were adjusted phenotypes obtained from fixed-effect correction rather than uncorrected raw records.

Phenotype adjustment was performed separately for each trait. Records with missing trait values or missing key environmental descriptors were removed. Obvious outliers were screened using the trait distribution within contemporary groups. A linear model including the available fixed effects described above was fitted, and the adjusted phenotype used for prediction was calculated as the residual plus the overall mean. This procedure ensured that Pearson correlations reflected genetic prediction performance rather than variation caused by herd, parity, age, season, or lactation-stage effects.

Whole-genome sequencing data with an average depth of approximately 1× were processed using a low-coverage sequencing workflow. Raw paired-end reads were filtered to remove adapter sequences and low-quality bases using Trimmomatic v0.39. Clean reads were aligned to the goat reference genome ARS1.2 (GCF_001704415.2) using BWA-MEM v0.7.15-r1140. The resulting alignment files were sorted and indexed using SAMtools v1.17, and duplicate reads were removed using Picard v2.25.5. Reference-panel genotypes were phased using Beagle v5.4, and genotype imputation of the approximately 1× target sequencing data was performed using GLIMPSE2 v2.0.0. GLIMPSE2 jointly used genotype likelihoods derived from the sequencing reads and phased reference haplotypes to infer posterior genotype probabilities and allele dosages, thereby accounting for uncertainty caused by low read depths. After imputation, variants were filtered according to the imputation quality and minor allele frequency, and the retained imputed genotypes were used for downstream genomic analyses.

Following genotype imputation, SNPs were filtered based on the imputation quality, minor allele frequency, and genotype missingness. The approximately one-million-marker dataset was constructed from the retained high-quality variants according to their physical positions on the ARS1.2 goat reference genome. To achieve approximately uniform genome-wide coverage, the number of SNPs assigned to each autosome was proportional to the chromosome length, and SNPs were selected at approximately equal physical intervals along each chromosome. When multiple variants were available near a target position, the SNP with the highest imputation quality was retained. In parallel, loci corresponding to a previously developed 25K SNP chip [11] were extracted from the same imputed dataset to construct a chip-based genomic dataset. The population structure and relatedness were evaluated using principal component analysis (PCA, PLink v1.90); these results were used to identify major population stratification and to support the interpretation of cross-validation performance.

### 2.2. Best Linear Unbiased Prediction Models

#### 2.2.1. Genomic Best Linear Unbiased Prediction (GBLUP)

Genomic best linear unbiased prediction (GBLUP) is a core method in quantitative genetics. The statistical model was specified under the following linear mixed-model framework:

*y* = 1*μ* + *Zg* + *e* (1)

where y is the vector of phenotypic observations, μ is the population mean, 1 is a vector of ones, g is the vector of genomic breeding values, Z is the incidence matrix linking records to genomic breeding values, and e is the residual vector. The key parameter is the genomic relationship matrix G, which was computed using the genome-wide marker algorithm proposed by VanRaden [12]:

(2) $$G=\frac{MM^{\prime }}{\sum_{l=1}^{m}2p_{j}q_{j}}$$

In this equation, M represents the standardized genotype matrix (with dimensions *n* × m, where *n* is the number of individuals and m is the number of SNP loci). $p_{j}$ represents the frequency of allele A1 at locus j, indicating the minor allele frequency at the j locus. The core assumption of this algorithm is that all SNP loci contribute equally to genetic covariance, thereby avoiding biases introduced by prior weight allocation. In the present study, GBLUP was implemented using the BGLR v1.1.0 package in R v4.3.0 [13].

#### 2.2.2. Ridge Regression Best Linear Unbiased Prediction (RR-BLUP)

The mixed-effects model based on ridge regression (RR-BLUP), as a pivotal method for genomic prediction, integrates the dual advantages of constrained regression and mixed linear models in its modeling process. Its statistical characteristics include ① its linear bias-free property, ensuring that the expected value of the estimator equals the true parameter value; ② the minimum prediction error variance criterion, guaranteeing estimator validity. The construction of its mathematical model adheres to the following principles:

(3) $$y=X?+Za+e,\;a\sim N\left(0,Is_{a}^{2}\right),s_{a}^{2}=\frac{s_{g}^{2}}{p}$$

RR-BLUP was implemented using the rrBLUP v4.6.1 package in R v4.3.0 [14].

### 2.3. Bayesian Regression Models

Bayesian regression follows the framework of Bayesian inference by treating model parameters as probability distributions rather than fixed values. The posterior distribution of each parameter is obtained by combining its prior distribution with the likelihood of the observed data. In genomic selection, Bayesian alphabet methods provide flexible models for marker effect estimation [15].

The BayesA method adopts a hierarchical Bayesian modeling strategy in which the parameter structure consists of two key levels. At the first level, marker effects are assumed to follow a Student’s t prior distribution with degrees of freedom v, which can be decomposed into a conditional normal distribution combined with a scale parameter. At the second level, the variance of marker effects is parameterized by an inverse chi-squared distribution, whose distributional properties are jointly determined by the hyperparameters S (scale parameter) and v (degrees of freedom). Unlike conventional mixture distributions, BayesA assumes a common prior distribution for all marker effects. Parameter estimation is performed within a Markov chain Monte Carlo (MCMC) framework, in which Gibbs sampling is used to iteratively sample from the conditional posterior distributions. In practice, parameter estimation and model fitting can be implemented using the BGLR package in R. The model is formulated as follows [16]:

(4) $$y=X\beta +\sum_{j=1}^{p}Z_{j}\alpha_{j}+e,\;\alpha_{j}∼N\left(0,\sigma_{\alpha_{j}}^{2}\right),\;\sigma_{\alpha_{j}}^{2}∼Inv-\chi^{2}\left(\nu ,S^{2}\right)$$

The BayesB method constructs a genetic dissection model with sparse characteristics. This algorithm handles marker effects through a two-stage probabilistic framework. First, the variance of marker effects is assigned a compound prior distribution, which consists of a mixture probability structure combining a discrete point-mass distribution and an inverse chi-squared distribution. Second, posterior sampling over the parameter space is performed using the Metropolis–Hastings algorithm. Unlike the homogeneous distributional assumption adopted in BayesA, BayesB assumes that only a proportion (1 − π) of the SNPs in the genome have non-zero effects, with their effect variances following an inverse chi-squared distribution, whereas the effects of the remaining proportion π of SNPs are constrained to zero. This sparse genetic architecture is achieved by introducing a binary selection mechanism in which the hyperparameter π is specified a priori to control the selection intensity of effective markers. In practice, parameter estimation can be conducted using the BGLR package in R [17].

(5) $$y=X\beta +\sum_{j=1}^{p}Z_{j}\alpha_{j}+e,\;where\;\alpha_{j}∼\{\begin{array}{c} N(0,\sigma_{\alpha_{j}}^{2}),with\;probability\;1-\pi \\ 0,with\;probability\;\pi . \end{array}$$

The BayesC method introduces probabilistic improvements to genomic selection models within a hierarchical Bayesian framework, with its major innovation being the hyperparameter modeling of the marker sparsity parameter (π). Compared with BayesB, in which π is treated as a fixed value, BayesC assumes (π~Uniform(0,1)), thereby enabling the data-driven and adaptive estimation of the sparsity level. In terms of model structure, BayesC retains the mixture distribution framework of BayesB, in which a subset of marker effects is assumed to follow an inverse chi-squared distribution, whereas the remaining marker effects are shrunk to zero. In addition, BayesC allows the effect variances of effective loci to be heterogeneous. Notably, its parameter updating procedure follows the Gibbs sampling framework used in BayesA. Parameter estimation is performed within an MCMC-based general linear mixed-model framework, which can be expressed as follows: [y = Xβ + Zα + ε], where (X) denotes the design matrix for fixed effects, (Z) denotes the marker effect matrix, (α) represents the vector of marker effects, and (ε) is the residual term. In practice, the computational implementation of BayesC can be carried out using the BGLR package in the R environment [18].

(6) $$y=X\beta +\sum_{j=1}^{p}Z_{j}\alpha_{j}+e,\;where\;\alpha_{j}∼\{\begin{array}{c} N(0,\sigma_{\alpha }^{2}),with\;probability\;1-\pi ,\\ 0,with\;probability\;\pi , \end{array}\;\pi ∼U(0,1)$$

The Bayes LASSO method was developed from the LASSO regression method proposed by Tibshirani, and its Bayesian formulation improves the predictive performance by introducing prior constraints on model parameters. Park and Casella established the core framework of this method within a Bayesian context, in which marker effects are assumed to follow conditional normal priors, with variance parameters assigned exponential distributions. Using a Gibbs sampling algorithm, the posterior distributions of both regression coefficients and variance parameters can be estimated simultaneously. In the field of genomic selection, the Bayesian LASSO has been shown to be statistically equivalent to the classical LASSO under certain conditions. Its hierarchical model is specified such that the variance of each SNP effect independently follows an exponential distribution, thereby shrinking most marker effects toward zero and consequently inducing model sparsity. This algorithm adopts a standard MCMC-based linear model structure, and parameter estimation can be implemented using the BGLR package in R [19].

(7) $$y=X\beta +\sum_{j=1}^{p}Z_{j}\alpha_{j}+e,\;\alpha_{j}∼Laplace(0,\lambda ),\;\lambda ∼Gamma(a,b)$$

### 2.4. Machine Learning Models

#### 2.4.1. Random Forest

Random forest (RF) is an ensemble learning algorithm that improves robustness by constructing multiple structurally diverse regression trees. Bootstrap resampling is used to generate M decision trees, each of which is trained independently on a subset of features. The final prediction is obtained by averaging the outputs of all trees, thereby reducing model variance:

(8) $$y=\frac{1}{M}\sum_{m=1}^{M}t_{m}\left(\psi_{m}(y:X)\right)$$

where y denotes the predicted value of the random forest regression model, and the predictor represents a decision tree constructed from a bootstrap sample of the data at the (m)-th iteration. (M) denotes the number of decision trees included in the random forest. During parameter optimization, a grid search strategy is used to determine the optimal value of (M). In this study, the RF model was constructed using the sklearn v1.3.0 package in Python v3.11.4 [20].

#### 2.4.2. Support Vector Regression

Support vector regression (SVR) is a regression extension of support vector machines and is grounded in statistical learning theory. By using non-linear kernels, SVR maps input data from the original space to a high-dimensional feature space, where non-linear relationships can be modeled. The prediction function can be expressed as

(9) $$f(x)=\beta_{0}+h{\left(x\right)}^{T}\beta$$

where *h*(*x*) denotes the kernel-induced feature mapping, which transforms the input data (x) into a high-dimensional feature space, and *β* represents the weight vector. Unlike the loss functions commonly used in traditional linear models, SVR adopts an insensitive loss function in which the loss is imposed only when the absolute difference between the predicted value and the observed value exceeds a predefined threshold. In this study, the SVR model was implemented using the sklearn package in Python [21].

#### 2.4.3. Kernel Ridge Regression

Kernel ridge regression (KRR) is a non-linear modeling method based on reproducing kernel Hilbert space theory [22]. It combines kernel methods with L2 regularization. Unlike ordinary ridge regression, KRR uses the kernel trick to define a high-dimensional feature space and then fits a ridge regression model in this space. The prediction model can be written as

(10) $$f(x)=k^{\prime }{\left(K+\lambda I\right)}^{-1}y$$

where K is the Gram matrix $K_{ij}=\langle \phi \left(x_{i}\right)\cdot \phi \left(x_{j}\right),k$; k is the vector $k_{i}=\langle \phi (x)\cdot \phi \left(x_{i}\right\rangle =k\left(x,x_{i}\right),i=$ 1,2,3,…,n,n of kernel similarities between a new individual and the training individuals; I is the identity matrix; and λ is the ridge parameter. Kernel functions and tuning parameters were selected by grid search. KRR was implemented in Python using scikit-learn to estimate individual GEBV values.

#### 2.4.4. Gradient Boosting Decision Tree

Gradient boosting decision tree (GBDT) is an ensemble learning method that iteratively reduces prediction errors by training a sequence of base learners, typically decision trees [23]. At each iteration, gradient information is used to update the model and correct the residual errors, thereby gradually improving the predictive performance of the model. The final GBDT model is expressed as the weighted sum of multiple base learners. When CART regression trees are used as the base learners, the resulting GBDT model can be regarded as a weighted combination of multiple multivariate piecewise functions. The prediction model of GBDT can be written as follows:

(11) $$F_{M}(x)=\sum_{m=1}^{M}\gamma_{m}h_{m}(x)$$

where $F_{M}(x)$ denotes the final prediction model; $h_{m}(x)$ represents the (m)-th base learner, usually a decision tree; and $\gamma_{m}$ is the weight assigned to the (m)-th base learner. GBDT constructs the model in an iterative manner. At each step (m), a new base learner $h_{m}(x)$ is added to minimize the loss function $L\left(y,F_{m-1}(x)+h_{m}(x)\right)$. In this study, the GBDT model was implemented using the scikit-learn package in Python.

#### 2.4.5. Light Gradient Boosting Machine

Light gradient boosting machine (LightGBM) is a decision tree-based gradient boosting algorithm. It reduces the loss function by sequentially adding decision trees, with each new tree designed to fit the residuals of the previous model [24]. Compared with other tree-based boosting methods, LightGBM uses a histogram algorithm and leaf-wise growth strategy, enabling faster training and lower memory consumption. For regression, the updating rule can be written as

(12) $$L=\sum_{i=1}^{n}{\left(y_{i}-{\hat{y}}_{i}\right)}^{2}$$

For binary classification, the predicted probability is often modeled as

(13) $$L=-\sum_{i=1}^{n}\left[y_{i}logp_{i}+\left(1-y_{i}\right)log\left(1-p_{i}\right)\right]$$

where $p_{i}=\frac{1}{1+e^{-y_{i}}}$ is the prediction probability.

Residual calculation: For t iteration round, the residual is $r_{i}^{\left(t\right)}=-{\frac{\partial L}{\partial {\hat{y}}_{i}^{\left(t-1\right)}}}^{7}$.

Tree model fitting: Build a decision tree $h_{t}(x)$, fit the residuals, and update predictions:

(14) $${\hat{y}}_{i}^{\left(t\right)}={\hat{y}}_{i}^{\left(t-1\right)}+\eta h_{t}\left(x_{i}\right)$$

where η is the learning rate. The final model integrates the weighted results of multiple decision trees:

(15) $${\hat{y}}_{i}=\sum_{t=1}^{T}\eta h_{t}\left(x_{i}\right)$$

In this study, LightGBM was implemented using the Python and sklearn software packages.

#### 2.4.6. Extreme Gradient Boosting

Extreme gradient boosting (XGBoost) is an ensemble learning algorithm based on gradient boosting decision trees. It improves standard GBDT by introducing regularization terms and second-order derivative information, thereby enhancing model performance and generalizability [25]. XGBoost is computationally efficient, supports parallel computing, uses pruning to reduce tree complexity, and approximates the loss function through Taylor expansion. It is widely used for classification, regression, ranking, anomaly detection, and model interpretation. The prediction is updated iteratively as follows:

(16) $${\hat{y}}_{i}^{\left(t\right)}={\hat{y}}_{i}^{\left(t-1\right)}+\eta f_{t}\left(x_{i}\right)$$

where η is the shrinkage parameter controlling the contribution of each tree, and $f_{t}\left(x_{i}\right)$ is the prediction from the t-th tree. The ensemble result from T trees is

(17) $${\hat{y}}_{i}=\sum_{t=1}^{T}\eta f_{t}\left(x_{i}\right)$$

In this study, XGBoost was implemented using the Python and sklearn software packages.

### 2.5. Generation and Integration of Prior Information

GWAS summary statistics and selection signature results were obtained from our previously published study [26]. SNPs were ranked according to the previously reported GWAS *p*-values, and the top 5%, 10%, 15%, and 20% of ranked SNPs were used to construct the GWAS-derived prior marker sets. Selection signature results were based on a comparison of 112 dairy goats and 64 non-dairy goats using the nucleotide diversity ratio, FST, Tajima’s D, and XP-CLR statistics. SNPs located within the top 1%, 2%, 3%, 4%, and 5% of previously identified candidate regions were extracted to construct the selection signature-derived prior marker sets.

### 2.6. Cross-Validation and Accuracy Assessment

Model performance was evaluated using 10-fold cross-validation. Individuals were randomly and evenly divided into 10 subsets; in each round, one subset was used as the validation set and the remaining nine subsets were used for model training. The machine learning models were fitted using the default parameter settings of the corresponding software implementations. No systematic hyperparameter search procedure was conducted. The validation subset was not used for model fitting. Prediction accuracy was assessed primarily using the Pearson correlation coefficient (PCC) between the predicted values and adjusted observed phenotypes.

(18) $${PCC}_{\left(y,y_{pre}\right)}=\frac{\sum_{i=1}^{N}\left(y_{i}-\bar{y}\right)\left(y_{i,pre}-\bar{y_{pre}}\right)}{\sqrt{\sum_{i=1}^{N}{\left(y_{i}-\bar{y}\right)}^{2}}\sqrt{\sum_{i=1}^{N}{\left(y_{i,pre}-\bar{y_{pre}}\right)}^{2}}}$$

Here, y_i_ and y_i,pre_ are the observed values of the i-th element, and ȳ and ${\bar{Y}}_{pre}$ are the mean values of y and y_pre_, respectively.

## 3. Results

### 3.1. Prediction Accuracy of Conventional Genomic Selection Models

**Figure 1:**
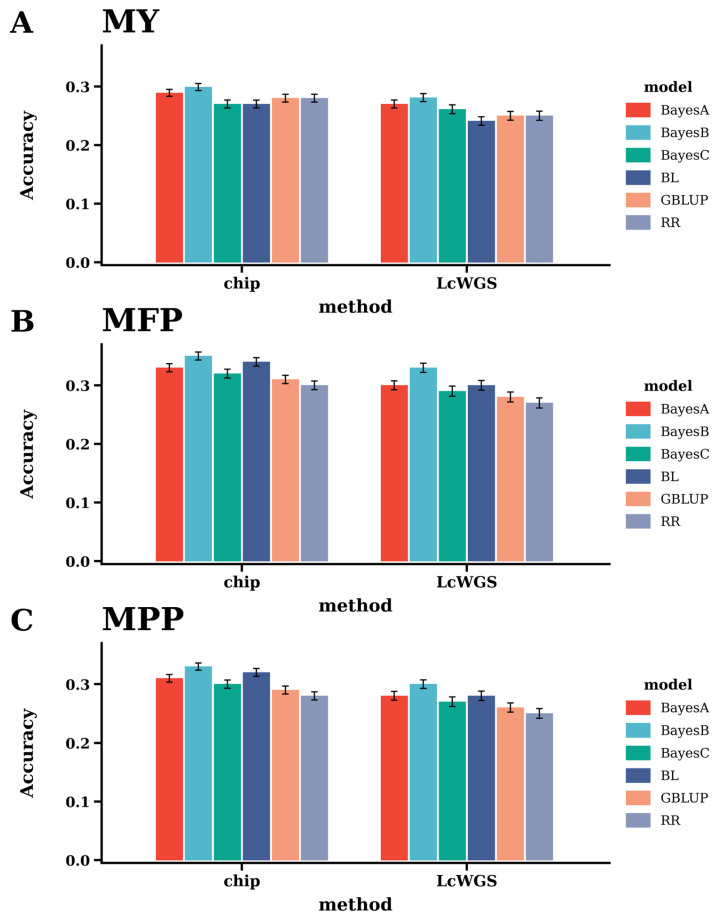
Predictive ability of conventional models for milk yield, milk fat percentage, and milk protein percentage. (**A**) Predictive ability for milk yield. (**B**) Predictive ability for milk fat percentage. (**C**) Predictive ability for milk protein percentage. MY, milk yield; MFP, milk fat percentage; MPP, milk protein percentage; BL, Bayesian LASSO; RR, ridge regression.

The systematic evaluation of conventional genomic selection models showed trait- and genotyping strategy-specific differences in prediction accuracy (Figure 1). Under the chip-based strategy, the highest PCC values were 0.30, 0.35, and 0.33 for MY, MFP, and MPP, respectively. For MFP, BayesB achieved a PCC of 0.35, compared with 0.31 for GBLUP, representing an improvement of approximately 12.9%. Under the lcWGS strategy, the highest PCC values were 0.28, 0.33, and 0.30 for MY, MFP, and MPP, respectively. For MY, BayesA improved the accuracy by approximately 3.6% compared with GBLUP under the chip-based strategy, whereas lcWGS reduced the overall prediction accuracy by 7.1%. For MPP, Bayesian LASSO improved the prediction accuracy by an average of 10.3% compared with the other Bayesian models. Across traits, lcWGS increased the prediction accuracy for MFP by 3.2% but reduced the prediction accuracy for MY by 6.8%.

### 3.2. Prediction Accuracy of Machine Learning Models

**Figure 2:**
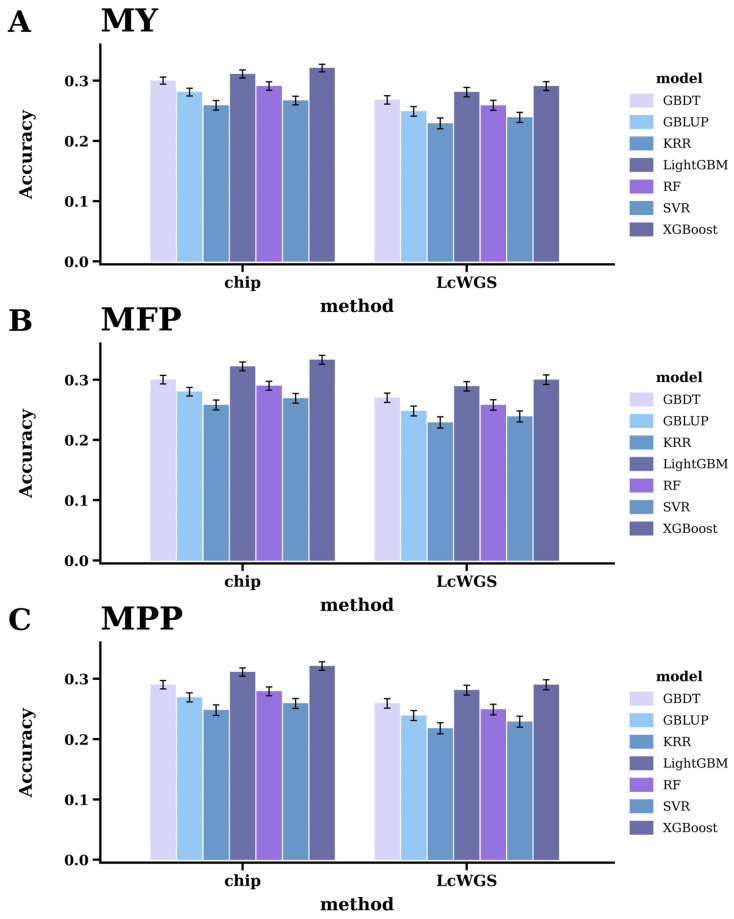
Predictive ability of machine learning models for milk yield, milk fat percentage, and milk protein percentage. (**A**) Predictive ability for milk yield. (**B**) Predictive ability for milk fat percentage. (**C**) Predictive ability for milk protein percentage. MY, milk yield; MFP, milk fat percentage; MPP, milk protein percentage.

Seven algorithms, namely GBDT, GBLUP, KRR, LightGBM, RF, SVR, and XGBoost, were evaluated using both chip and lcWGS data (Figure 2). Under the chip-based strategy, XGBoost showed the strongest overall performance, achieving PCC values of 0.32, 0.33, and 0.32 for MY, MFP, and MPP, respectively. The corresponding PCC values obtained using GBLUP were 0.28, 0.28, and 0.27. Thus, XGBoost improved the prediction accuracy by 14.3%, 17.9%, and 18.5% for MY, MFP, and MPP, respectively, compared with GBLUP. Under lcWGS, LightGBM improved MFP prediction by 6.7% relative to the chip-based strategy but reduced the MY prediction accuracy by 9.7%. KRR showed favorable performance for MPP under lcWGS, with prediction accuracy that was 12.1% higher than that of RF, although its accuracy for MY decreased by approximately 7.4%. SVR showed relatively stable performance across the two genotyping strategies.

### 3.3. Genomic Selection Based on GWAS Prior Information

**Figure 3:**
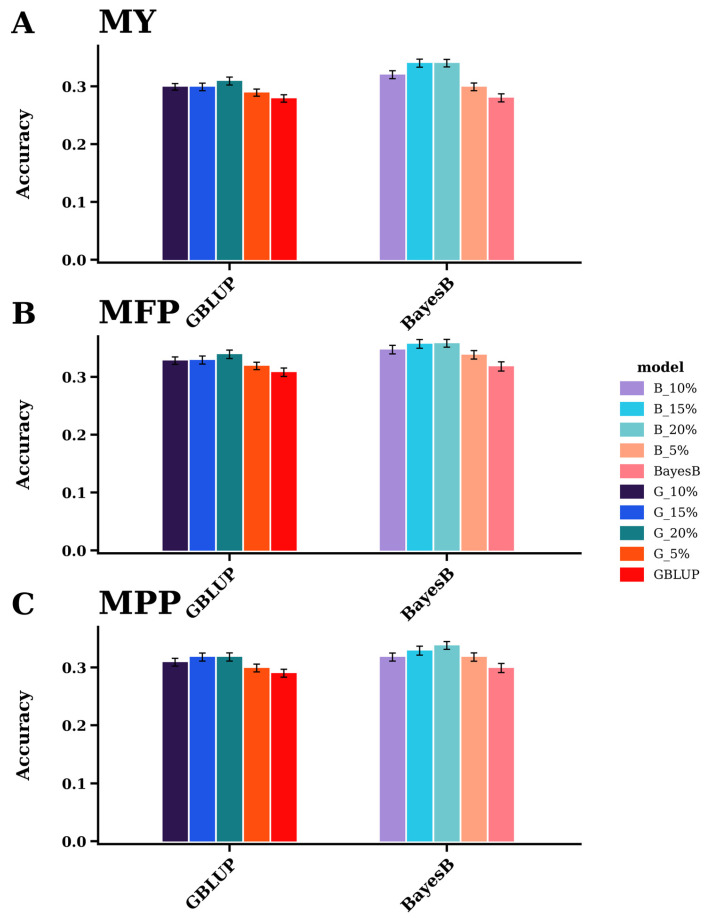
Predictive ability of genomic selection based on GWAS prior information. (**A**) Predictive ability for milk yield using different models with GWAS prior information. (**B**) Predictive ability for milk fat percentage. (**C**) Predictive ability for milk protein percentage. MY, milk yield; MFP, milk fat percentage; MPP, milk protein percentage.

GWAS-derived prior information at different top-ranking proportions (top 5–20%) was incorporated into the chip data, and the predictive performance of GBLUP and BayesB was evaluated for MY, MFP, and MPP (Figure 3). For MFP, incorporating the top 20% GWAS priors improved BayesB’s performance by approximately 12.9% relative to the baseline model and by 9.7% compared with GBLUP when using the same proportion of priors. For MY, integrating the top 15% GWAS priors improved BayesB’s performance by 6.5% relative to GBLUP, whereas the improvement decreased to 4.5% when the prior proportion increased to 20%. For MPP, GBLUP and BayesB showed the smallest difference when the top 10% GWAS priors were included. Overall, BayesB showed an accumulated improvement of approximately 23.1% across the top 10–20% prior information range for MFP, whereas GBLUP showed an improvement of 14.3% across the same range.

### 3.4. Genomic Selection Based on Selection Signature Prior Information

**Figure 4:**
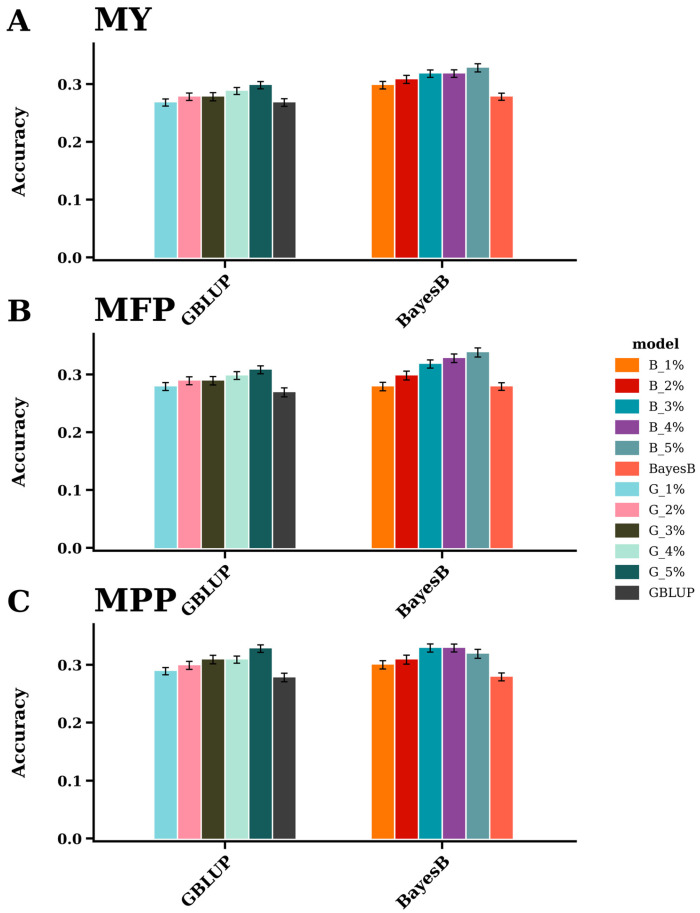
Predictive ability of genomic selection based on selection signature prior information. (**A**) Predictive ability for milk yield using models incorporating selection signature priors. (**B**) Predictive ability for milk fat percentage. (**C**) Predictive ability for milk protein percentage. MY, milk yield; MFP, milk fat percentage; MPP, milk protein percentage.

Selection signature prior information (top 1–5%) was integrated with chip data to evaluate GBLUP and BayesB’s prediction performance for dairy goat milk production traits (Figure 4). For MY, BayesB improved the prediction accuracy by approximately 10.7% when the top 3% priors were incorporated, while the improvement was 7.1% at the top 5% level. GBLUP showed a cumulative improvement of 9.5% across the same prior information range. For MFP, BayesB achieved a 13.8% relative improvement over GBLUP when the top 5% priors were integrated. For MPP, GBLUP reached its highest performance when the top 2% priors were included, improving the accuracy by 10.7% relative to the baseline model. BayesB reached its maximum improvement at the top 5% level and outperformed GBLUP by 9.1% at the same proportion. Overall, BayesB showed cumulative improvements of 7.1%, 15.2%, and 9.1% for MY, MFP, and MPP, respectively.

## 4. Discussion

This study systematically evaluated genomic prediction for dairy goat milk production traits and showed that the predictive ability was influenced by the model type, genotyping strategy, and integration of biological prior information. The results demonstrate the practical value of comparing multiple model classes and marker sources when designing genomic selection strategies for dairy goats.

Conventional genomic prediction models and machine learning methods provide complementary information for genetic evaluation [27]. Among the conventional models, BayesB performed particularly well for MFP, which is consistent with previous studies showing that Bayesian variable selection models can be effective for traits influenced by heterogeneous marker effects [28,29]. These results support the use of Bayesian approaches as useful alternatives to linear genomic prediction models for milk composition traits.

Gradient boosting algorithms also showed strong predictive performance, especially XGBoost under the chip-based strategy. The improved PCC values for MY, MFP, and MPP indicate that ensemble learning methods can effectively model complex marker patterns and contribute to genomic prediction in dairy goats. Similar advantages of machine learning methods have been reported in genomic prediction studies of other livestock populations, supporting their value as flexible tools for breeding applications [30,31].

The comparison between chip-based and lcWGS marker sets further highlighted the importance of matching the genotyping strategy to the target traits. The chip-based dataset provided strong predictive ability for MY, whereas lcWGS was informative for some milk composition traits. This pattern suggests that the marker density, marker selection, and trait architecture jointly determine the efficiency of genomic prediction and that both SNP chip and lcWGS strategies can be valuable depending on the breeding objective [32,33,34,35,36].

The integration of GWAS-derived prior information improved the predictive ability for several trait–model combinations. The favorable response of BayesB for MFP indicates that SNPs with stronger association signals can provide useful information for Bayesian prediction models. Because the GWAS prior markers were ranked by their association *p*-values, this strategy provides a straightforward way to incorporate statistical evidence from previous association analyses into genomic selection [37].

Selection signature priors also improved prediction for selected traits, suggesting that genomic regions shaped by artificial or natural selection can contribute useful biological information to genomic prediction. The use of dairy goat versus non-dairy goat comparisons, together with the FST, nucleotide diversity ratio, Tajima’s D, and XP-CLR statistics, provided a biologically informed marker prior strategy. These results support the integration of selection signature information as a promising approach in improving the genomic prediction of dairy goat milk traits [38,39,40].

From an implementation perspective, conventional linear and Bayesian models remain attractive because their assumptions, computational requirements, and breeding value outputs are familiar to routine genetic evaluation programs. Tree-based boosting methods can improve the predictive ability, but they require additional hyperparameter tuning, greater computational resources, and careful control for validation leakage. Therefore, the highest predictive accuracy should not be considered in isolation. A customized 25K-plus-prior panel may offer a practical compromise among genotyping and imputation cost, computational burden, prediction accuracy, and ease of deployment, but its value should ultimately be judged by whether it changes the selection rankings and increases the expected genetic gain in a commercial breeding program.

This study also has several limitations. The reference population was relatively modest and consisted of dairy goats sampled from specific Chinese production regions, which may limit its transferability to other breeds, management systems, and countries. The prediction results were obtained by internal cross-validation and require confirmation in independent dairy goat populations. In addition, the current manuscript does not quantify sample overlap or close relatedness between the present population and the previous GWAS population, does not include equally sized random marker benchmarks, and does not compare the concordance of top-ranked selection candidates across models and marker panels. These analyses are important in determining whether prior-informed gains reflect biological prioritization and whether they materially affect breeding decisions.

Overall, this study establishes a practical model–data–prior framework for dairy goat genomic selection. The results indicate that BayesB is useful for milk composition traits, XGBoost is effective under the chip-based strategy, and GWAS- or selection signature-informed marker sets can further enhance the predictive ability. Future studies integrating larger multi-farm populations, additional validation cohorts, and multi-omics information will further strengthen the application of these models in precision dairy goat breeding.

## 5. Conclusions

This study compared conventional genomic prediction models, Bayesian regression models, and machine learning algorithms for milk production traits in dairy goats using SNP chip and lcWGS marker datasets. The results demonstrated that model performance was trait-dependent and closely related to the genotyping strategy. BayesB showed advantages for milk composition traits, whereas XGBoost achieved strong predictive ability under the chip-based strategy. Incorporating GWAS-derived and selection signature prior information further enhanced the prediction performance, especially for milk composition traits. These results highlight the value of combining statistical models, machine learning methods, and biological prior information to improve genomic prediction in dairy goats, and they provide a practical framework for the precision breeding of dairy goat milk production traits.

## Data Availability

The genotype and phenotype data are not deposited in a public repository because they contain farm and breeding program information. De-identified data and the analysis scripts may be made available by the corresponding author upon reasonable request, subject to institutional approval and applicable data sharing restrictions.
